# Development of a Pulsatile Flow-Generating Circulatory Assist Device (K-Beat) for Use with Veno-Arterial Extracorporeal Membrane Oxygenation in a Pig Model Study

**DOI:** 10.3390/biology9060121

**Published:** 2020-06-12

**Authors:** Yutaka Fujii, Nobuo Akamatsu, Yasunori Yamasaki, Kota Miki, Masayuki Banno, Kenta Minami, Shuji Inamori

**Affiliations:** 1 Department of Clinical Engineering and Medical Technology, Niigata University of Health and Welfare, Niigata 950-3198, Japan; 2 Division of Clinical Engineer, Department of Medical Technology, Osaka City General Hospital, Osaka 534-0021, Japan; ce_nobu3@icloud.com; 3 Department of Medical Engineering, Faculty of Health Sciences, Aino University, Ibaraki 567-0012, Japan; y-yamasaki@me-u.aino.ac.jp; 4 Department of Medical Engineering Center, Ehime University Hospital, Toon, Ehime 791-0204, Japan; ce_ecc_kota_m@yahoo.co.jp (K.M.); banno.masayuki.fn@ehime-u.ac.jp (M.B.); 5 Department of Clinical Engineering, Japan Community Healthcare Organization, Osaka Hospital, Osaka 534-0021, Japan; minami-kenta@osaka.jcho.go.jp; 6 Department of Clinical Engineering, Junshin Gakuen University, Fukuoka 815-0036, Japan; inamori.s@junshin-u.ac.jp

**Keywords:** K-beat, pulsatile, diastolic augmentation, myocardial, V-A ECMO

## Abstract

Veno-arterial extracorporeal membrane oxygenation (V-A ECMO) preserves the life of heart failure patients by providing an adequate oxygen supply and blood flow to vital organs. For patients with severe cardiogenic shock secondary to acute myocardial infarction or acute myocarditis, V-A ECMO is commonly used as the first choice among cardiac circulatory support devices. While V-A ECMO generates circulatory flow using a centrifugal pump, the provision of pulsatile flow is difficult. We previously reported our development of a new circulatory flow assist device (K-beat) for cardiac management with pulsatile flow. To obtain more efficient pulsatile assist flow (diastolic augmentation), an electrocardiogram (ECG)-analyzing device that can detect R waves and T waves increases the assist flow selectively in the diastole phase by controlling (opening and closing) the magnetic valve of the tamper. Here, we describe the first use of the K-beat on a large animal in combination with a clinical device. In addition, the diastolic augmentation effect of the K-beat as a circulatory flow assist device was examined in a pig V-A ECMO model. The K-beat was stopped every 60 min for a period of a few minutes, and blood pressure waveforms in the pulsatile and non-pulsatile phases were checked. This experiment showed that stable V-A ECMO could be achieved and that hemodynamics were managed in all animals. The pulsatile flow was provided in synchrony with the ECG in all cases. A diastolic augmentation waveform of femoral arterial pressure was confirmed in the pulsatile phase. K-beat could be useful in patients with severe heart failure.

## 1. Introduction

Veno-arterial extracorporeal membrane oxygenation (V-A ECMO) preserves the life of patients with heart failure by providing an adequate oxygen supply and blood flow to vital organs [1]. Furthermore, for patients with severe cardiogenic shock secondary to acute myocardial infarction or acute myocarditis, V-A ECMO is commonly used as the first-choice cardiac circulatory support device [1,2,3,4,5]. In recent years, pulsation caused by a centrifugal pump has been described [6]. However, the control is difficult and requires modifications of the device. Such intervention is far from commonplace. In the human body, the pulsatile circulatory flow is generated by the heart. A pulsatile flow pattern is important not only to ensure an efficient circulatory flow throughout body but also to maintain optimal end-organ function. Loss of pulsatility is associated with neurological complications and peripheral circulatory failure [7,8]. To maintain pulsatile flow during total cardiopulmonary bypass, intra-aortic balloon pumping (IABP) can be used, but the insertion of the balloon is associated with additional surgical stress. In addition, the use of IABP while maintaining the patient’s own heartbeat is expected to have a diastole augmenting effect. Such diastolic augmentation would increase the oxygen supply to the myocardium, but the surgical procedure of IABP insertion is essential.

We previously reported our development of a new circulatory flow assist device (K-beat) for cardiac management with pulsatile flow [9]. K-beat is very simple and requires no additional surgical procedure. K-beat is composed of a V-A ECMO system with a portable magnetic valve device, which consists of a pulse generator and a tamper that produces intermittent mechanical compressions, attached to the pillow of the sending tube. The magnetic valve of the tamper is controlled by a pulse generator that synchronizes pulsatile flow with the patient’s heart rate [9]. To obtain more efficient pulsatile assist flow (diastolic augmentation), an electrocardiogram (ECG)-analyzing device detects R waves and T waves to increase the assist flow selectively in diastole by controlling (opening and closing) the magnetic valve of the tamper. ECG synchronization is controlled using a relay circuit with an operational amplifier [8]. Here, we describe the first experiment conducted using a combination of an actual clinical device and the K-beat in a large animal study. In addition, the efficiency of the K-beat as a circulatory flow assist device was examined in a pig V-A ECMO model by focusing on effects of diastolic augmentation. The diastolic augmentation effect increases coronary flow by actively increasing aortic pressure during diastole.

## 2. Materials and Methods

### 2.1. Ethical Approval

This study was conducted with the approval of the Animal Care and Use Committee at Osaka City General Hospital (ethics approval code: 2016–22). All procedures met the standards of the National Institutes of Health guidelines for animal care. Male pigs (body weight, 25–35 kg) were housed one per cage under a 12 h light-dark cycle with food and water available ad libitum.

### 2.2. Anesthesia, Surgical Preparation, and V-A ECMO

Pigs were anesthetized with intramuscular atropine (0.04 mg/kg), tiletamine HCl/zolazepam HCl (4.0 mg/kg), and xylazine (4.0 mg/kg). The pigs were placed in a supine position with ECG electrodes and a rectal thermocouple in place. Rectal temperature was maintained at 36 °C throughout the experiment. Orotracheal intubation was then performed using a size 6.0 cuffed endotracheal tube, and animals were ventilated with a respirator (ANESTHESIA APPARATUS, Model D-5F, ACE3000; Acoma Medical Industry, Tokyo, Japan). Ventilation was volume-controlled at a frequency of 15 breaths/min and a tidal volume of 10 mL/kg body weight. Anesthesia was maintained by inhalation of isoflurane (2.0–3.0%) administered in 50–60% oxygen. Arterial blood pressure was monitored (Model 870, Power Lab system; AD Instruments, Castle Hill, Sydney, Australia) via the femoral artery, which was cannulated with 4-Fr polyethylene tubing (Atom Medical Corporation, Tokyo, Japan). To access the ascending aorta and heart, median sternotomy was performed. A 16-Fr cannula (PAA061CB, Edwards, Tokyo, Japan) was placed in the ascending aorta to serve as the arterial inflow cannula for the V-A ECMO circuit. Heparin sodium (500 IU/kg) was administered after placement of this cannula. A 20-Fr cannula (TFM020L; Edwards) was placed in the right atrium to serve as a conduit for venous outflow. Generally, the first choice is to place the inflow and outflow cannula at peripheral sites. However, in this study, a central approach was used for V-A ECMO for anatomical reasons (the femoral artery is thin).

The V-A ECMO circuit consisted of a membranous oxygenator (CX-LX2MW; Terumo, Tokyo, Japan), a tubing line (JUNKEN MEDICAL Co., Tokyo, Japan), and a centrifugal pump (CX-SP4538H; Terumo) primed with 480 mL of Ringer’s solution.

### 2.3. Experimental Design

**Figure 1:**
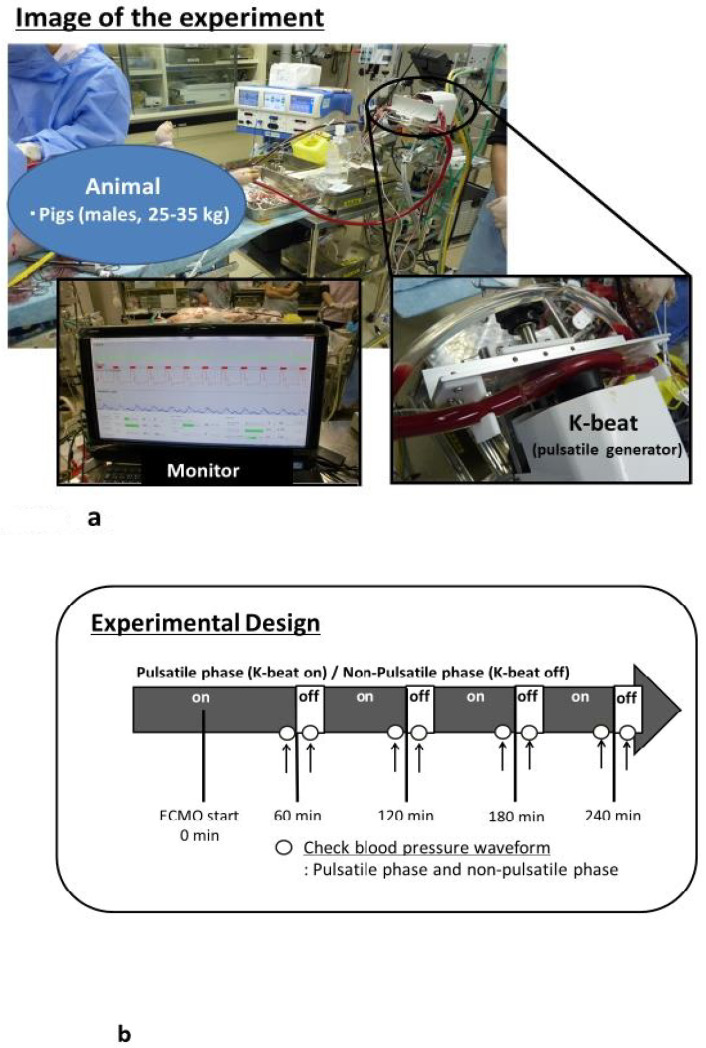
(**a**) Photograph of the experimental set-up. (**b**) Experimental design for pulsatile flow (K-beat on), and non-pulsatile flow (K-beat off).

Three male pigs (body weight 25–35 kg) were placed under V-A ECMO support. V-A ECMO was maintained for 240 min, with a pump flow of 50–60 mL/kg/min. Arterial pressure of carbon dioxide (PaCO_2_) and arterial pressure of oxygen (PaO_2_) were maintained at 35–45 mm/Hg and 200–300 mm Hg, respectively. Mean arterial pressure was maintained above 70 mm/Hg. Animals received heparin at 500 IU/kg for systemic anticoagulation. The target activated coagulation time was 200–250 s. The K-beat was stopped every 60 min for a period of a few minutes, and blood pressure waveforms in the pulsatile and non-pulsatile phases were checked. Figure 1 shows the experimental set-up and protocol design (Figure 1a,b). All animals were sacrificed at the end of V-A ECMO by potassium chloride injection into the heart.

## 3. Results

**Figure 2:**
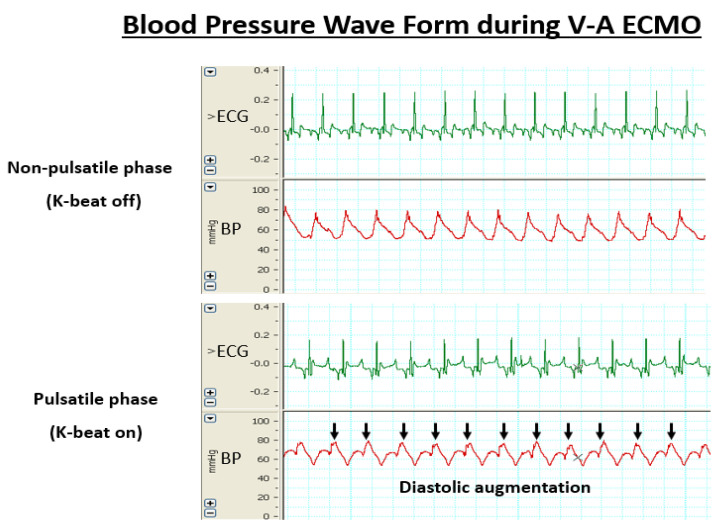
Blood pressure waveform during V-A ECMO. Upper: Non-pulsatile phase (K-beat on). Lower: Pulsatile phase (K-beat off). Black arrow indicates increased diastolic pressure.

A stable V-A ECMO along with the management of hemodynamics was able to be provided in all animals. In all cases, pulsatile flow was produced in synchrony with the ECG. Table 1 shows changes in hemodynamic variables, pH, hemoglobin (Hb) concentration, and levels of electrolytes during experiments. Figure 2 shows representative examples of femoral artery waveforms during V-A ECMO (pulsatile and non-pulsatile phases). A diastolic augmentation waveform was confirmed in the pulsatile phase.

## 4. Discussion

In all cases, pulsatile flow was able to be produced in synchrony with the ECG. Generally, IABP is used in combination with V-A ECMO to provide pulsatile flow [10]. With our new pulsatile flow-generating circulatory assist device (K-beat), IABP is not required. The use of K-beat is thus associated with reduced surgical risk, is less time-consuming, and is simple to set up for patients in emergency situations.

In this study, a diastolic augmentation waveform was confirmed in the pulsatile phase. This is expected to increase myocardial perfusion. We are currently performing measurements of myocardial blood flow using a laser Doppler blood flow meter (Laser Doppler ALF21; ADVANCE Co., Tokyo, Japan) in the same experimental system. At this point, an increasing trend is seen in the amount of myocardial blood flow in the pulsatile phase. Therefore, during this time, diastolic augmentation was confirmed from the arterial pressure wave form. On the other hand, we could not clearly observe the systolic unloading effect.

Further animal experiments will be needed to clarify the utility of this device. No electrolyte abnormalities were identified from blood data. In a previous study, we performed V-A ECMO for 48 h using a similar system [9]. No damage was found to the inner surface of the ECMO circuit and pillow in that previous experiment [9]. However, further investigations are needed to clarify the safety of the K-beat pulsatile flow generator system and blood cell damage during large animal V-A ECMO. This study had several limitations. First, a thick cannula was inserted into the aorta, but in actual clinical practice, the approach would be via peripheral vessels. Second, the arrhythmia detection function and issues of safety (including device durability) have yet to be examined. These issues need careful consideration in the future. In addition, in recent years, a pump that can provide pulsatile flow has been used (e.g., i-cor diagonal pump; Xenios AG, Heilbronn, Germany) [6,11,12]. In the future, comparisons will also need to be made with results from the i-cor pump. We would also like to verify the effects of pulsatile flow with the K-beat when used in cardiac arrest.

## 5. Conclusions

In conclusion, this study demonstrated that the K-beat pulsatile flow-generating circulatory assist device could be used with V-A ECMO in a pig model. This device provided pulsatile flow in synchrony with the ECG. A diastolic augmentation waveform was confirmed in the pulsatile phase. K-beat could prove useful in patients with severe heart failure.

## Figures and Tables

**Table 1:** Hemodynamic variables, pH, Hb, and levels of electrolytes before and during veno-arterial extracorporeal membrane oxygenation (V-A ECMO).

		HR	MAP	Hb	pH	Na	K	Cl
		(beats/min)	(mm/Hg)	(g/dL)		(mEq/L)	(mEq/L)	(mEq/L)
Case 1	pre	87	68	11.2	7.356	141	4.3	106
	240 min	85	70	9.2	7.352	142	5.3	106
Case 2	pre	80	73	12.9	7.401	138	4.6	110
	240 min	82	71	8.2	7.361	140	5.4	108
Case 3	pre	75	64	10.7	7.387	138	3.9	110
	240 min	79	67	7.4	7.346	139	5.2	109

HR: heart rate; MAP: mean arterial pressure; Hb: hemoglobin.

## Abbreviations

The following abbreviations are used in this manuscript:

V-A ECMO	Veno-arterial extracorporeal membrane oxygenation
ECG	Electrocardiogram
IABP	Intra-aortic balloon pumping
PaO2	Arterial pressure of oxygen
PaCO2	Arterial pressure of carbon dioxide
